# Non-Surgical Rhinoplasty After Nasal Skin Cancer Reconstruction: Enhancing Esthetic Outcomes

**DOI:** 10.3390/jcm14155394

**Published:** 2025-07-31

**Authors:** Shahin Tahan Shoushtari, Charles Savoldelli, Héloïse Gobillot, Laurent Castillo, Gilles Poissonnet, Philippe Kestemont, Grégoire D’Andréa, Clair Vandersteen

**Affiliations:** 1 Head and Neck Departement, Institut Universitaire de la Face et du Cou, 31 Avenue de Valombrose, UCA, 06300 Nice, France; 2 Head and Neck Departement, Institut Universitaire de la Face et du Cou, 31 Avenue de Valombrose, UR2CA-REBOOT, UCA, 06300 Nice, France; 3 Geriatrics Department, Pitié-Salpêtrière, Sorbonne Université, 47-83 Boulevard de l’Hopital, 75013 Paris, France

**Keywords:** non-surgical rhinoplasty, hyaluronic acid, nasal reconstruction, quality of life

## Abstract

**Objectives:** Nasal reconstructive surgery following skin cancer resection is challenging, with esthetic concerns impacting patients’ quality of life. Non-surgical rhinoplasty may be an alternative to repeated surgeries. This study aimed to evaluate non-surgical rhinoplasty esthetic benefits and subjective patient outcomes after skin cancer resection. **Methods:** We conducted a retrospective study on patients with post-operative esthetic dissatisfaction after nasal skin cancer surgery, who underwent non-surgical rhinoplasty with hyaluronic acid. Subjective benefits were evaluated with the FACE-Q Rhinoplasty self-questionnaire at three consultations: before injection (baseline), and at one and two months after. Two-dimensional and three-dimensional Vectra H2 photographs were used to assess subjective esthetic concerns and objective volumetric changes. **Results:** The study included six female patients with an average age of 58.3 years. They had undergone, on average, five nasal surgeries for cancer. The mean FACE-Q scores were 53.3 (±10.31), 77.5 (±4.18), and 79.7 (±6.76), respectively, at baseline, one month, and two months. Significant differences were observed between baseline and one month (*p* < 0.001) and between baseline and two months (*p* < 0.001), but not between one and two months. The was a mean volumetric gain of 1.13 mL at one month and 1.19 mL at two months. **Conclusions:** This preliminary study suggested that hyaluronic acid-based non-surgical rhinoplasty could improve esthetic outcomes and quality of life in patients who had undergone nasal skin cancer surgery. These findings highlight a potential role for this minimally invasive technique in selected post-reconstructive cases, although the small sample size limited the generalizability of the results and underlined the need for further prospective evaluation.

## 1. Introduction

Skin cancer is the most prevalent form of cancer, affecting individuals of all genders. Their initial treatment typically involves surgical removal, sometimes extensive, followed by customized reconstruction. Tumor-free margins remain one of the main prognostic factors, particularly for basal cell carcinomas (BCCs) and squamous cell carcinomas (SCCs), which account for up to 95.4% of these cancers [1]. Face tumor location can significantly impact both esthetic and functional surgical outcomes. Due to lifelong and increasing exposure to solar radiation, the skin of the nose is particularly prone to the development of these UV-induced skin tumors [2,3], representing 25.5% of facial tumor sites [4].

For these patients, the esthetic and functional consequences of surgery can have profound social and psychological impact, potentially leading to gradual social isolation following remission [5,6,7,8], because the nose is central to facial identity and non-verbal communication; even subtle defects can therefore translate into pronounced disturbances in self-image, professional interactions, and intimate relationships. Post-operative management may include procedures to improve esthetics. However, these are sometimes delayed due to concerns about oncological control, patient reluctance, or uncertainty about meeting the patient’s expectations.

Recent advances in esthetic injection procedures, coupled with the refinement of practitioners’ skills, provide a high level of safety against potential complications [9], particularly with regard to the use of fillers in nasal reconstructive surgery [10]. The specific rheological properties and ease of use of materials extensively studied in primary and secondary non-surgical rhinoplasty could complement the tools available to facial reconstruction surgeons, who aim to improve both the esthetic and functional outcomes of their interventions.

In parallel, the recent development of three-dimensional (3D) photographic technologies offers a valuable complement to subjective assessments of post-procedural esthetic outcomes, enabling the objective visualization and quantification of morphological changes in patients [11]. These systems are particularly suited for the evaluation of volumetric facial treatments, such as hyaluronic acid injections, by providing reproducible and quantifiable data that enhance outcome analysis [12].

The aim of this study was to evaluate the perceived benefit of non-surgical rhinoplasty in enhancing the esthetic outcomes of reconstructive surgery for nasal skin cancer, as well as to objectively quantify the volumetric gain provided by this technique using 3D imaging.

## 2. Materials and Methods

### 2.1. Patients

We conducted a retrospective study on patients who had undergone non-surgical rhinoplasty with hyaluronic acid injections following surgery to remove nasal skin cancer and who reported dissatisfaction with esthetic outcomes. All included patients had been treated (surgery and injection) in the same center, between May 2015 and March 2023. Eligibility criteria included a history of nasal skin carcinoma treated surgically with reconstruction, a minimum of one year since the last oncologic surgery, a patient-expressed desire for esthetic improvement, and either refusal of further surgery or a context in which surgical revision was uncertain or not recommended. Exclusion criteria included any standard contraindications to hyaluronic acid injections, oncologic surgery performed less than one year prior or with uncertain resection margins, metastatic evolution, and cases where surgical correction remained more appropriate and accessible than injection-based management. We extracted clinical data from their electronic health records, which included age, tumor histology, the number of surgical procedures they underwent, the kind of reconstruction performed, the time elapsed from the last oncological surgery and reconstructive surgery, and the planned treatment for non-surgical rhinoplasty. This study was recorded in an official clinical registry with the Public Health Department’s Informatics and Liberties Committee under registration number 2024-EI-621. As a purely retrospective study, the work received an exemption and a formal waiver from the Institutional Review Board. All procedures were conducted in accordance with the latest version (October 2013) of the Declaration of Helsinki.

### 2.2. Non-Surgical Rhinoplasty

This procedure was performed after securing informed consent from the patients. This included providing them with an explanation of the expected benefits and risks associated with the injection of hyaluronic acid. Restylane^®^ Lyft (Galderma, Lausanne, Switzerland) was used for all procedures. A pre-hole was created with a needle, and the rhinoplasty was performed using a 25-gauge cannula following a single-point injection technique [13]. To break post-operative adhesions from the reconstructive surgery, successive movements with the cannula were required. Upon completion of the procedure, a manual massage was performed to evenly distribute the product across the targeted areas. A one-month reflection period was observed between the initial consultation and the actual procedure. Data collection took place before injection, as well as one and two months post injection.

### 2.3. Subjective Outcomes

At the start of each consultation, patients filled out the FACE-Q Rhinoplasty “Nose Satisfaction” questionnaire, which had been validated in French [14]. The score was normalized on a scale of 100. Data from the statistical analysis were expressed as means ± standard deviations and medians with an interquartile range of 25 to 75%. Differences in FACE-Q scores between each consultation were analyzed using a paired-samples *t*-test (or Student’s *t*-test) with R Studio version 2023.06.0+421. Results were considered significant for an alpha risk level of α ≤ 0.05.

### 2.4. Imaging Methodology

Traditional 2D photographs were taken using a standard reflex camera. Three-dimensional photographs were captured using the Vectra H2 system (Canfield Scientific, Parsippany, NJ, USA) paired with an EOS^®^ Rebel T8i camera. This system acquired three successive images—frontal, right three-quarter, and left three-quarter views—which were then automatically merged by the Vectra^®^ software (version 7.4.17) to create a precise three-dimensional facial model. The system provided a spatial resolution of approximately 1 mm, with reported intra- and inter-operator reproducibility below 2%, ensuring reliable detection of subtle morphological changes over time [15].

By aligning two 3D reconstructions obtained at different time points, the software calculated the displacement vectors for each vertex of the mesh, enabling analysis of local volume changes. Areas of volume gain (positive projection) were visualized in shades of blue, while areas of volume loss or secondary traction (negative projection), typically resulting from the expansion of adjacent regions, were shown in shades of red.

For quantitative analysis, a diamond-shaped region of interest was defined, bounded superiorly by the nasion, laterally by the projection of the alar bases onto the nasolabial folds, and inferiorly by the anterior nasal spine. The software then computed the mean volumetric gain within this region, providing an objective measure of augmentation.

The consent and image release form was consistently signed.

### 2.5. Subjective Peer Assessment by Oncodermatology Specialists

The final photographic results, along with pre-treatment images, were shown to five practitioners who specialized in facial reconstructive surgery and were not familiar with the use of skin injectables in their daily clinical practice. Each practitioner evaluated the esthetic outcomes using a Likert scale. The scores were as follows: 0 (no benefit), 1 (minimal benefit), 2 (moderate benefit), 3 (significant benefit), and 4 (major benefit). At the end of the evaluation, the practitioners were asked to answer the following question: “Do these results encourage you to consider training and practicing hyaluronic acid injections as a supplement to your nasal reconstructions?”

## 3. Results

1. Population

Six female patients were included, with a mean age of 58.3 years. The primary indication for non-surgical rhinoplasty was post-operative reconstruction following skin squamous cell carcinoma. The average time between the last oncological surgery and hyaluronic acid injections was 36.5 months, while the average time between the last reconstructive surgery and injections was 11 months. Before undergoing non-surgical rhinoplasty, patients had already undergone an average of 4.5 surgical procedures, including carcinoma excision, reconstruction, and other corrective surgeries. Five patients achieved a satisfactory result with a 1 mL hyaluronic acid injection, while one patient required 2 mL. No complications occurred after these injections. The clinical characteristics of these patients are reported in Table 1.

2. FACE-Q outcomes

All patients completed the FACE-Q evaluation scale, specifically the Nose Satisfaction section. This allowed for the assessment of the subjective esthetic benefit of non-surgical rhinoplasty at baseline, one-month, and two-month time points. The mean scores were 53.3 (±10.31) at baseline, 77.5 (±4.18) at one month, and 79.7 (±6.76) at two months. The detailed results are presented in Table 2.

**Figure 1:**
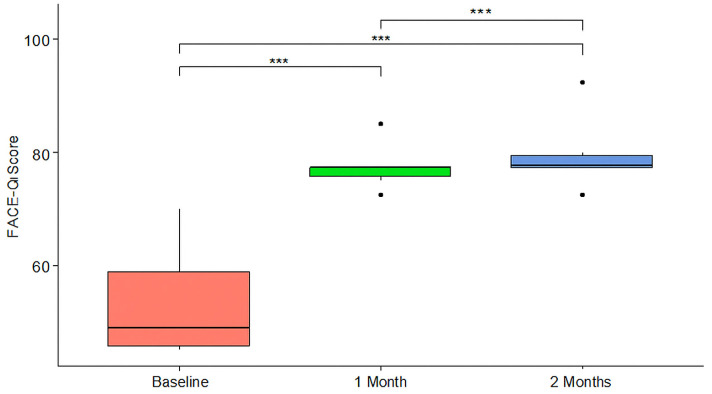
Boxplots of the distribution of FACE-Q Scores at different time points. *** = *p* value < 0.001.

**Figure 2:**
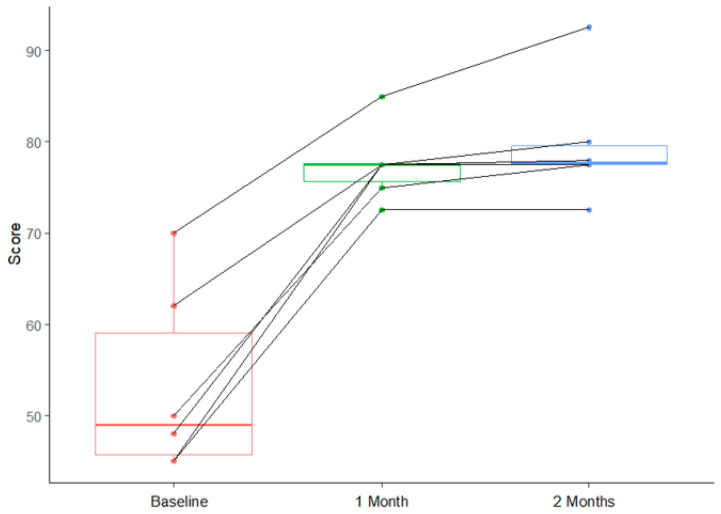
Individual trajectories of FACE-Q scores from baseline to one and two months.

The difference in scores between baseline and one month was significant (*p* < 0.001), as was the difference between baseline and two months, but there was no significant difference between one month and two months. All results were reported with 95% confidence intervals to reflect the precision of the estimated differences. These results are shown in Table 3 and modeled with a box plot in Figure 1. The improvement in overall scores for each patient at one and two months was illustrated in Figure 2.

3. Specialist evaluation and volumetric outcomes

The average hetero-evaluation scores reported by practitioners following traditional photograph assessments two months after non-surgical rhinoplasty reached 2.7 on a scale of 4. All practitioners affirmed that these findings had a significant impact on their decision to integrate hyaluronic acid injections into their oncodermatology practices.

**Figure 3:**
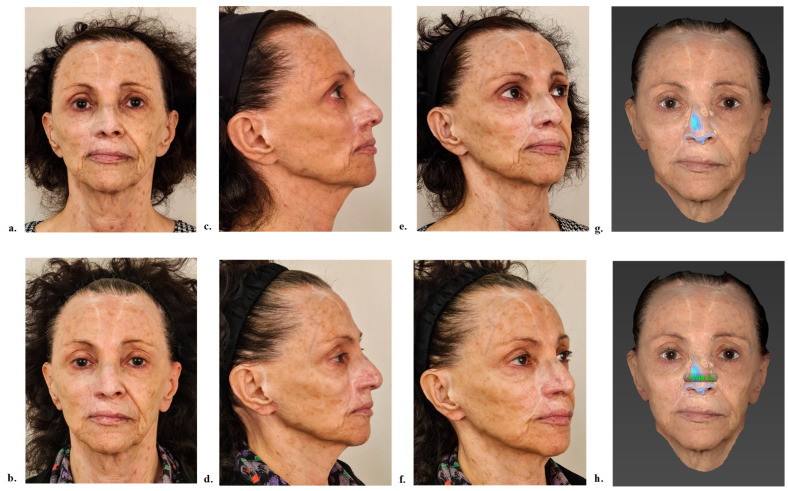
Standardized photographs of patient 5 before (**a**,**c**,**e**) and two months after (**b**,**d**,**f**) hyaluronic acid injection: front view (**a**,**b**), profile view (**c**,**d**), and oblique view (**e**,**f**). Visualization of projection vectors on the nasal pyramid using Vectra software version 7.4.17 (**g**) and calculation of volumetric changes (**h**), with a volume gain of +1.44 cc in the blue-delineated area.

Volumetric gains were calculated using standardized 3D photographs acquired with the Vectra^®^ H2 imaging system (Canfield Scientific, Parsippany, NJ, USA). The 3D images were reconstructed and analyzed using the associated software and were presented alongside their corresponding traditional photographic views for visual comparison, as illustrated in Figure 3.

**Figure 4:**
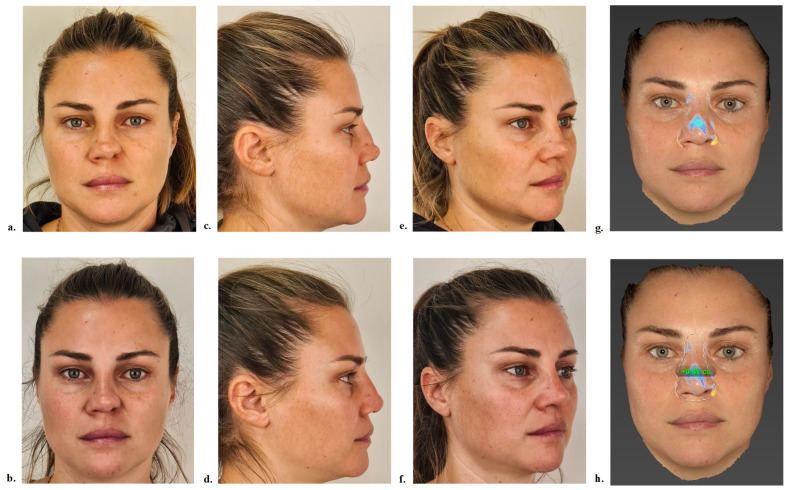
Standardized photographs of patient 4 before (**a**,**c**,**e**) and two months after (**b**,**d**,**f**) hyaluronic acid injection: front view (**a**,**b**), profile view (**c**,**d**), and oblique view (**e**,**f**). Visualization of projection vectors on the nasal pyramid using Vectra software version 7.4.17 (**g**) and calculation of volumetric changes (**h**), with a volume gain of +0.42 cc in the blue-delineated area.

**Figure 5:**
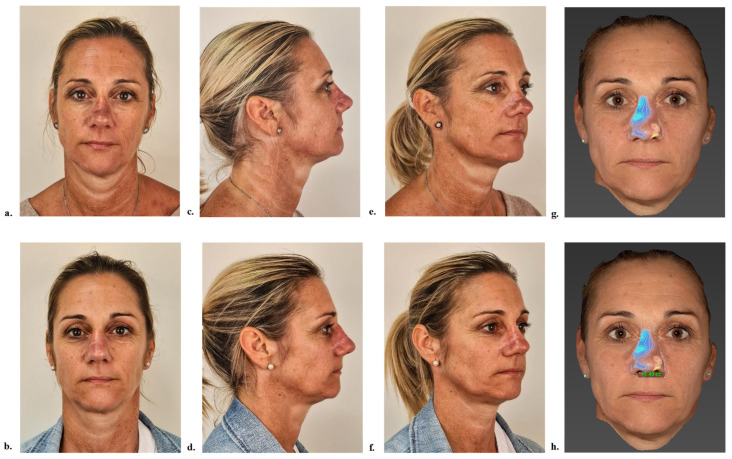
Standardized photographs of patient 6 before (**a**,**c**,**e**) and two months after (**b**,**d**,**f**) hyaluronic acid injection: front view (**a**,**b**), profile view (**c**,**d**), and oblique view (**e**,**f**). Visualization of projection vectors on the nasal pyramid using Vectra software version 7.4.17 (**g**) and calculation of volumetric changes (**h**), with a volume gain of +1.49 cc in the blue-delineated area.

At one month and two months post-injection, the average volumetric gains were 1.13 mL and 1.19 mL, respectively. Across the cohort, individual volume gains ranged from 0.42 mL for the lowest observed correction (Figure 4) to 1.49 mL for the largest (Figure 5).

## 4. Discussion

Since Beer’s groundbreaking publication in 2006 [16], which suggested using hyaluronic acid as an alternative to surgical rhinoplasty, and Nyte’s 2007 study outlining its application in treating internal valve collapse [17], non-surgical rhinoplasty has experienced a surge in popularity and a broadening in its indications. Whether performed as a primary procedure on a non-operated nose or as a secondary post-operative approach [18], it has become a crucial tool, often used in conjunction with botulinum toxin [19], to enhance the esthetic and functional outcomes of rhinoplasty and improve patient satisfaction [20]. In an oncological context, nasal reconstruction is a complex process, with outcomes influenced by the extent of initial tissue damage, the nature of the procedure performed, and the unique characteristics of each patient. This type of surgery, which is now well-documented, is known for its complexity [21]. The flap techniques used and the challenge of accurately recreating the delicate nasal anatomy, which contributes to both its esthetics and physiology, can require multiple interventions [22,23]. Frequently, multiple surgical stages are necessary, and additional revision surgeries may be proposed or requested. In a meta-analysis of 1334 cases, Rohrich et al. found that 25% of patients needed more than two surgeries during their reconstruction journey [24]. Given the high incidence of skin tumors on the nasal pyramid, a significant number of patients undergo these procedures. With recent advances in esthetic medicine, these patients may benefit from the post-operative use of alternative injectable techniques to enhance quality of life after reconstructive surgery. This aspect sometimes becomes deprioritized once the neoplastic condition is under control.

To the best of our knowledge, this study represents the first subjective and objective account of non-surgical rhinoplasty performed following nasal reconstruction in a post-operative oncological context. In this study, some patients had undergone as many as 10 surgeries. The impact of these surgeries on patients’ psychological and emotional states can be underestimated. Several studies on patients’ quality of life with cutaneous melanomas, squamous cell carcinomas, and basal cell carcinomas demonstrate the significant impact of these surgeries, particularly when the initial lesion is aggressive and the treatment is disfiguring. Patients in our study were younger compared to other studies (58.3 years vs. 66.7 years [1]), possibly indicating a group more concerned with esthetic outcomes. Vaidya et al. identified younger age, nasal surgery, reconstruction by local flap, and a history of anxiety or depression as independent risk factors for postoperative psychosocial distress using the FACE-Q Skin Cancer Appearance-Related Psychosocial Distress scale [25]. These risk factors correspond to those seen in our cohort, which comprises mainly younger female patients who underwent complex nasal reconstructions with local flaps. For such at-risk individuals, offering a straightforward, non-surgical, and low-cost option—such as hyaluronic acid rhinoplasty—can provide meaningful psychological support by showing that esthetic improvement remains attainable after oncologic and reconstruction treatment.

The FACE-Q Rhinoplasty module, a rhinoplasty-specific subscale of the FACE-Q Aesthetics suite, is used to assess patient-reported outcomes. This questionnaire, composed of ten items specifically addressing nasal appearance, is now a recognized and widely used tool in rhinoplasty research. Since its introduction in 2010, it has become increasingly used in both surgical and non-surgical rhinoplasty, reflecting the growing interest in Patient-Reported Outcome Measures (PROMs) within the field of esthetic medicine [26]. For the present study, we used the validated French translation of the instrument, thereby ensuring linguistic and cultural appropriateness for our patient cohort [14]. Although the measure captures inherently subjective judgements, its structured format produces quantitative scores that permit reliable comparisons across interventions and time points. Several recent publications on nonsurgical rhinoplasty have demonstrated the questionnaire’s sensitivity to change [27] and its suitability for evaluating volumetric augmentation techniques [28]. Accordingly, incorporating the FACE-Q Rhinoplasty questionnaire allowed a rigorous, standardized assessment of patient satisfaction following our procedure.

Our findings highlight patient satisfaction following reconstruction-focused non-surgical rhinoplasty. The emphasis these patients place on achieving esthetic results that restore their quality of life and approximate their pre-cancer appearance may significantly contribute to the elevated satisfaction rates measured by FACE-Q. While the surgical outcomes may seem satisfactory given the extensive tissue damage from the carcinologic excision and the complexity of reconstruction, it is crucial to acknowledge the potential for wide variability in patients’ perceptions. After cancer treatment, even minor deformations can act as psychological reminders of the initial trauma. When surgical results plateau after multiples procedures, the provision of a non-surgical solution that can be performed during a consultation and holds medico-socio-economic interest can offer an unexpected efficient benefit due to its straightforward implementation. This satisfaction appears to persist for up to two months, but longer-term evaluation will require additional studies.

The volumetric calculation, conducted using the Vectra H2 system and its associated software, enabled an objective and automatic quantification of the volumetric gain induced by hyaluronic acid injections. The incorporation of high-resolution 3D stereophotogrammetry represents a methodological advance over exclusive standardized photographs. By capturing sub-millimeter surface alterations, 3D imaging bridges the gap between patient-reported satisfaction and objective tissue response, providing a precise and reproducible measurement tool for clinicians and researchers alike [15,29]. In the specific context of volumizing agents, this technology offers an opportunity to characterize dose–response relationships, map diffusion planes, and optimize product selection according to anatomical sub-units of the nose. It is important to note that the volume administered does not directly correlate with the esthetic quality of the outcome. However, this analysis is particularly valuable in validating the shifts and stability of volume over time in the targeted areas, as delineated in the patient’s treatment plan. The characteristic durability of hyaluronic acid varies across non-surgical rhinoplasty studies, ranging from 1 to 8 years [30], reinforcing its potential in long-term management. The effect of fibrosis after treatment and the low mobility of the nasal pyramid may explain these persistent results. Further longer studies are needed to assess product stability in the nose.

Moreover, our findings suggest that the use of hyaluronic acid helps contribute to skin trophicity improvement, as suggested by patients’ standardized photographs. The benefits of skin hydration and the release of adhesion areas were universally reported by all patients during follow-up consultations. These observations align with previous research papers that have highlight the significant impact of hyaluronic acid on skin hydration and volume restoration, owing to its ability to stimulate collagen and elastin production (key components in maintaining skin elasticity and structure) [31,32,33].

The spread of hyaluronic acid in the realm of esthetic medicine since the 1990s has been reflected in the escalating number of PubMed database publications dedicated to this topic. Its use to enhance the results of cancer surgery has raised concerns about its safety with respect to neoplastic diseases. Studies investigating the relationship between hyaluronic acid and tumor proliferation have confirmed the product’s safety, as described in the conclusive report from the Cosmetic Ingredient Review (CIR) program in 2016 [34]. Additionally, this compound has been studied as a carrier for local chemotherapy in the treatment of skin carcinomas [35]. In an effort to mitigate risks, our study maintained a rigorous interval of at least one year following the last oncological surgical intervention. Furthermore, the product chosen for the injections was specifically researched in non-surgical rhinoplasty to ensure its safety [36]. Alternative techniques such as autologous fat grafting, cartilage onlays, or secondary debulking procedures may also improve nasal contours. However, these methods typically require access to the operating room, involve more invasive procedures, and are associated with longer recovery times. Fat grafting, although potentially effective, can be unpredictable in previously operated areas due to scarring and adhesions, which may hinder graft integration and volume stability. Cartilage grafting requires harvesting from a donor site—often the ear or septum—introducing additional morbidity [37], and necessitates surgical dissection of the nasal region, including re-elevation of tissues, which increases procedural complexity. These surgical options are generally more durable over time but come with greater patient burden [38]. In contrast, hyaluronic acid injections can be performed during routine medical consultation, with minimal procedural burden and rapid recovery. While this technique does not aim to replace surgical revision when feasible and reliable, it offers a valuable alternative when further surgery is either contraindicated or carries high uncertainty regarding the esthetic outcome.

Despite the absence of any complications in this study, it is imperative to acknowledge that non-surgical rhinoplasty, akin to any procedure involving hyaluronic acid injection, carries inherent risks. The complexity and variability of nasal vascularization warrant careful consideration [39]. Of particular concerns is the presence of an anastomosis between the facial artery and the internal carotid system via the angular artery [40], which poses the risk of blindness due to retro-embolization into the ophthalmic artery [41]. However, such events are rare, especially with the use of blunt-tip cannulas across the nasal pyramid. In a recent 2024 meta-analysis encompassing 9657 nonsurgical rhinoplasty procedures, Song et al. reported a severe complication rate of 0.27% and found no significant difference in the risk of vascular occlusion between blunt-tip cannulas and sharp needles. The authors emphasized the importance of withdrawal aspiration and thorough anatomical knowledge in minimizing complications [42,43,44]. By contrast, the 2020 meta-analysis by Harb et al., based on 5000 procedures, documented 24 vascular occlusion events—three of which resulted in localized necrosis—all occurring during nasal tip injections performed with needles [45]. These findings suggest a potentially higher risk with sharp instrumentation, although the role of injection technique versus instrument type remains subject to ongoing debate. In these rare circumstances, the administration of hyaluronidase, the antidote for hyaluronic acid, is crucial [46]. Rouanet et al. outlined a detailed protocol in 2022 for addressing a range of potential scenarios [47]. Its availability represents an additional advantage of the injectable approach due to its reversibility. It provides a safeguard that surgical cartilage grafts or autologous fat transfers cannot offer [48]. This pharmacological controllability may further reassure both surgeons and patients who are reluctant to undergo additional invasive procedures [49,50,51].

Finally, this study aimed to assess not only the subjective satisfaction of patients but also the interest in these techniques among surgeons specialized in oncodermatology. The results are favorable toward this technique, suggesting potential for the integration of esthetic medicine as a valuable an asset for these practitioners. The benefits, as evaluated from photographs, indicate that these techniques could serve as a valuable complement to surgery for these practitioners.

While the use of botulinum toxin has been explored in various reconstructive contexts, its application in nasal reconstruction remains relatively under-investigated. Preliminary evidence suggests that preoperative injection of botulinum toxin may enhance wound healing by reducing muscular tension and improving vascular conditions at the surgical site [52,53]. These effects—combined with its influence on inflammation and fibroblast activity [54,55]—have been associated with better scar quality in selected facial procedures. However, its specific role in nasal pyramid reconstruction has not yet been systematically studied. Investigating its potential to improve both esthetic and functional outcomes in this context represents a promising direction for future research.

Beyond hyaluronic acid, the integration of other esthetic medical tools could further optimize outcomes for patients with persistent nasal deformities after cancer surgery. A combined approach using botulinum toxin—to modulate perinasal muscle activity—and suspension threads—to restore tissue support—alongside hyaluronic acid injections, could address a broader range of post-reconstructive imperfections [56]. Such multimodal strategies could offer significant benefits, particularly in cases where surgical options have been exhausted. A prospective study evaluating these complementary techniques could provide deeper insight into their synergistic potential and further define the role of esthetic medicine in post-oncologic facial rehabilitation.

The main limitation of this study was the small sample size, which reduces the statistical robustness of the analyses. Although esthetic medicine has been integrated into surgical rhinoplasty protocols, its application in oncodermatology cases remains relatively recent. As a result, not all patients under long-term follow-up have been informed of these enhancement options in cases of persistent esthetic dissatisfaction. To strengthen the conclusions of this study, a larger-scale prospective study will be necessary. In this regard, the present work serves as an exploratory evaluation of the outcomes achievable in this specific indication. Given the encouraging results, we are currently planning a prospective study with defined inclusion criteria—namely, patients who have undergone nasal oncologic surgery and express esthetic concerns that could be addressed through non-surgical techniques. Such a design would allow for a more rigorous assessment of both esthetic and quality-of-life outcomes, with long-term follow-up extending to at least one year.

Moreover, to ensure optimal and lasting results, the structural integrity of the underlying nasal framework appears to be a key determinant in the efficacy of hyaluronic acid injections. This is particularly illustrated by patient 4, who required two syringes of filler due to a marked saddle nose deformity secondary to partial nasal septum resection. The lack of adequate support has likely contributed to product migration, highlighting the limitations of fillers in structurally compromised noses. In such cases, a thorough pre-injection assessment is essential to anticipate the need for increased product volume, or even to reconsider the suitability of a non-surgical approach.

## 5. Conclusions

This preliminary study indicates that hyaluronic acid-based non-surgical rhinoplasty can meaningfully enhance both esthetic outcomes and health-related quality of life in patients who have undergone nasal skin cancer surgery. These encouraging results also introduce a fresh therapeutic perspective for reconstructive surgeons and the physicians responsible for long-term follow-up: a minimally invasive option that can address persistent esthetic concerns in cases where surgical reconstruction is complete or no longer considered feasible. Given the limited sample size and retrospective nature of this work, these results should be interpreted as an initial exploration. Confirmation of these preliminary findings will require larger, prospective cohorts with extended follow up and comprehensive outcome assessment.

## Figures and Tables

**Table 1:** Surgical characteristics of the population. SCC = squamous cell carcinoma. BCC = basal cell carcinoma. T-stage = tumor stage. R0 = tumor-free margins.

	Age (years)	Histological Type T-Stage and Margin Status	Number of Surgeries	Time Since Last Oncologic Surgery (Months)	Time Since Last Cosmetic Surgery (Months)	Reconstruction	Injection Target
Patient 1	77	SCC; T2; R0	1	30	30	Rintala flap	Saddle nose correction
Patient 2	64	BCC; T1; R0	3	23	3	Skin graft	Graft expansion
Patient 3	62	SCC; T3; R0	7	48	10	Bilateral frontal flap	Nasal tip projection
Patient 4	37	Melanoma; T2; R0	4	100	93	Cartilage graft and local flaps	Saddle nose correction
Patient 5	68	SCC; T3; R0	10	43	6	Frontal and nasolabial flaps	Dorsum symetrisation
Patient 6	42	BCC; T2; R0	2	12	12	Double nasolabial flaps	Dorsum projection

**Table 2:** Distribution of means, medians, and dispersion of FACE-Q scores at different time points. SD = standard deviation. IQR = interquartile range.

FACE-Q Score	Baseline (n = 6)	1 Month (n = 6)	2 Months (n = 6)
Mean (±SD)	53.33 (±10.31)	77.50 (±4.18)	79.67 (±6.76)
Median [IQR]	49.00 [45.75–59.00]	77.50 [75.62–77.50]	77.75 [77.50–79.50]
Minimum	45.00	72.50	72.50
Maximum	70.00	85.00	92.50

**Table 3:** Mean differences in FACE-Q scores between consultations. Values in parentheses represent 95% confidence intervals. No missing data, N = 6. Mean differences between scores at different times were tested using a Student’s *t*-test for paired data.

Compared Consultations.	Mean Differences with 95% CI	*p* Value
Baseline vs. 1 Month	24.17 (16.47; 31.86)	<0.001
Baseline vs. 2 Months	26.33 (19.51; 33.16)	<0.001
1 Month vs. 2 Months	2.17 (−0.83; 5.17)	0.122

## Data Availability

The data are not publicly available due to privacy restrictions.
